# Determination and Visualization of pH Values in Anaerobic Digestion of Water Hyacinth and Rice Straw Mixtures Using Hyperspectral Imaging with Wavelet Transform Denoising and Variable Selection

**DOI:** 10.3390/s16020244

**Published:** 2016-02-18

**Authors:** Chu Zhang, Hui Ye, Fei Liu, Yong He, Wenwen Kong, Kuichuan Sheng

**Affiliations:** 1 College of Biosystems Engineering and Food Science, Zhejiang University, Hangzhou 310058, China; chuzh@zju.edu.cn (C.Z.); yehui03@zju.edu.cn (H.Y.); kcsheng@zju.edu.cn (K.S.); 2 School of Information Engineering, Zhejiang A&F University, Hangzhou 311300, China; wwkong16@zafu.edu.cn

**Keywords:** hyperspectral imaging, anaerobic digestion, pH value, distribution map, variable selection

## Abstract

Biomass energy represents a huge supplement for meeting current energy demands. A hyperspectral imaging system covering the spectral range of 874–1734 nm was used to determine the pH value of anaerobic digestion liquid produced by water hyacinth and rice straw mixtures used for methane production. Wavelet transform (WT) was used to reduce noises of the spectral data. Successive projections algorithm (SPA), random frog (RF) and variable importance in projection (VIP) were used to select 8, 15 and 20 optimal wavelengths for the pH value prediction, respectively. Partial least squares (PLS) and a back propagation neural network (BPNN) were used to build the calibration models on the full spectra and the optimal wavelengths. As a result, BPNN models performed better than the corresponding PLS models, and SPA-BPNN model gave the best performance with a correlation coefficient of prediction (*r_p_*) of 0.911 and root mean square error of prediction (RMSEP) of 0.0516. The results indicated the feasibility of using hyperspectral imaging to determine pH values during anaerobic digestion. Furthermore, a distribution map of the pH values was achieved by applying the SPA-BPNN model. The results in this study would help to develop an on-line monitoring system for biomass energy producing process by hyperspectral imaging.

## 1. Introduction

With the development of society, economy and technology, fossil fuels have been greatly exploited and used. An urgent issue is that the fossil fuels are non-renewable energy, and fossil fuels will run out in the near future. In recent years, biomass has been used for energy production and represents a huge supplement for meeting the energy demand [1,2,3].

The use of biomass energy can contribute to sustainable development, and reduce greenhouse gas emissions [2,3]. The sources of biomass energy are often locally available and easy to get. Vegetal biomasses are important sources of biomass energy [2,3]. Studies have been reported the use of different plant materials as biomass energy resources [2,3,4,5,6]. Anaerobic digestion is the main procedure for producing biomass energy [7,8,9,10]. Moreover, the pH value is one of the major factors influencing the anaerobic digestion performance and final biomass energy yield [11,12,13]. During the dynamic process of anaerobic digestion, the pH values vary during different digestion stages. Nonetheless, an optimum pH value of the reaction liquid would result in a higher yield of biomass energy. Generally, pH values are determined by a pH meter or pH sensors, which can accurately measure pH values. However, those methods are difficult to operate, and require sample contact and precalibration. Those methods can only determine the pH value of the sensor location spot, and cannot measure the pH of every spot within a certain area. With the development of intelligent process control, a rapid and contactless method should be developed.

Near-infrared spectroscopy has been applied as a contactless, fast and easy to operate method to monitor the dynamic process of anaerobic digestion. Stockl, *et al*. used near-infrared spectroscopy to monitor the anaerobic digestion process and determine the concentrations of organic acids [14]. Krapf, *et al.* successfully used a near infrared (NIR) spectroscopy online process analyser developed from the laboratory to monitor *in situ* the volatile solids (VS), ammonium, total inorganic carbon, and volatile fatty acids parameters of anaerobic digestion of energy crops and livestock residues [15]. Kandel, *et al*. used near infrared reflectance spectroscopy and PLS to predict the specific biogas yield (SBY), specific methane yield (SMY) and kinetics of biogas yield (k-SBY) of reed Canary grass (RCG) biomass [16].

As a technique integrating both spectroscopic technology and imaging technology, hyperspectral imaging has gained continuous attention from different fields. Hyperspectral imaging provides both the spectral and spatial information simultaneously. Each pixel within the hyperspectral image contains a spectrum at the spectral range of the system, and there is a gray-scale image at each wavelength. The advantage of hyperspectral imaging makes it possible to predict the parameters of every pixel within the image during the dynamic process of anaerobic digestion. Hyperspectral imaging has been utilized to visualize the distribution of quality parameters for different samples [17,18,19,20]. The application of hyperspectral imaging to anaerobic digestion would be helpful to comprehensively monitor the anaerobic digestion process and control the parameters that influence the anaerobic digestion for the better production of biomass energy. Hyperspectral imaging has been applied in the field of anaerobic digestion. Bonifazi, *et al*. used a hyperspectral imaging system to monitor in real time total solid (TS), volatile solid (VS), chemical oxygen demand (COD), and ammonia nitrogen (NH_4_-N) content of anaerobic digestion of different plants, with satisfactory results [21]. However, to our knowledge, no studies on the application of hyperspectral imaging to monitor the pH value during the anaerobic digestion have been reported.

The main objective of this study was to explore the feasibility of determining and visualizing the pH value of the anaerobic digestion liquid of water hyacinth and rice straw mixtures by hyperspectral imaging. The specific objectives were: (1) to build linear and non-linear models for pH value determination; (2) to select optimal wavelengths by different methods for the pH value determination; (3) to form the distribution maps of the pH value of the anaerobic digestion liquids.

## 2. Materials and Methods

### 2.1. Sample Preparation

The anaerobic digestion liquids for methane production were produced from rice straw and water hyacinth. The rice straw was collected from the paddy fields from Shangyun (Zhejiang, China). The collected rice straw was naturally air dried and ground into particles with a diameter of 5 mm. The ground particles were stored until further use. The water hyacinth was collected from the Xixi Wetland, Hangzhou (Zhejiang, China). The collected water hyacinths were washed and checked to remove any rotten parts, then dehydrated and naturally air dried. The dried water hyacinth was ground into particles with a diameter of 5 mm. The ground particles were stored until further use.

The inoculum sludge for the anaerobic digestion was collected from Hangzhou Zhengxin Animal Husbandry Ltd. (Hangzhou, China). The water hyacinth particles were mixed with the rice straw particles at a ratio of 3:1. Then the mixture was mixed with the inoculum sludge and water. Each day during the anaerobic digestion, the anaerobic digestion liquids were collected and placed in Petri dishes (three samples per day) and stored in a −80 °C refrigerator. At the end of the experiments, these samples were thawed, then the pH value measurement and hyperspectral image acquisition were conducted simultaneously. In total, 93 samples were collected for this purpose.

### 2.2. Hyperspectral Imaging System and Image Acquisition

A near-infrared hyperspectral imaging system covering the 874–1734 nm spectral range was used. The hyperspectral imaging system consisted of an imaging spectrograph (ImSpector N17E; Spectral Imaging Ltd., Oulu, Finland), a CCD camera (Xeva 992; Xenics Infrared Solutions, Leuven, Belgium) equipped with a camera lens (OLES22; Specim, Spectral Imaging Ltd., Oulu, Finland). The system was placed in a darkroom with two 150 W tungsten halogen lamps (3900 Lightsource, Illumination Technologies Inc; New York, NY, USA) for illumination and a conveyer belt driven by a stepper motor (Isuzu Optics Corp, Taiwan) for sample movement. The anaerobic digestion liquids were put into Petri dishes, and the Petri dishes were placed on the conveyer belt for image acquisition.

### 2.3. Hyperspectral Image Acquisition and Correction

The acquired images were raw images and needed to be corrected with white and dark reference images. The white reference image was captured using a white Teflon tile with nearly 100% reflectance. The dark reference image was captured by turning off the light source and covering the camera lens completely with its opaque cap with nearly zero reflectance. The correction of the hyperspectral images was conducted according to the following equation:

(1) $$I_{c}=\frac{I_{r}-I_{d}}{I_{w}-I_{d}}$$

where *I_r_* was the raw image, *I_w_* was the white reference image and *I_d_* was the dark reference image.

### 2.4. Spectral Data Extraction

For each Petri dish, the region that covers the Petri dish without the edge was selected as the region of interest (ROI) and all pixels within the ROI were extracted and preprocessed. The average spectrum of the preprocessed spectrum of each pixel within the ROI was calculated as the sample spectrum.

### 2.5. Measurement of pH Vvalue

The pH value of the samples was measured by a pH meter (Testo 205, Schwarzwald, Germany) after the image acquisition.

### 2.6. Chemmometric Methods

#### 2.6.1. Spectra Preprocessing

The extracted spectrum of each pixel was preprocessed before averaging due to the random noises of the spectrum of each pixel. Wavelet transform (WT) has been used as an efficient method to reduce spectral noises. WT decomposes the spectra into the high frequency parts and low frequency parts with different wavelet basis functions and different decomposition levels. The high frequency parts containing the noises are preprocessed by the threshold method. The wavelet basis function and decomposition level are crucial to the preprocessing performance [22].

#### 2.6.2. Calibration Methods

Partial least squares is the most used method for multivariate analysis of spectral data. It explores the linear relationship between the spectral data and the chemical or physical features, and compresses the spectral data into new orthogonal variables (called latent variables, LVs). The first few LVs carrying the most useful information are used for calibration [23]. In this study, the optimal number of LVs was determined by the software Unscrambler^®^ 10.1 (CAMO AS, Oslo, Norway).

Back propagation neural network (BPNN) is a widely used multilayer feedforward neural network. It consists of an input layer, a hidden layer and an output layer. BPNN has a good nonlinear mapping ability. BPNN continuously adjusts the weights and thresholds of the network by the feedforward of errors. In this study, a three layer BPNN model was used. The determination of the number of neurons in the hidden layer is important for any BPNN model. The number of neurons in the hidden layer was determined by comparing the performance of BPNN models using different number of hidden neurons. The number of neurons in the hidden layer to be selected could be based on the following equation:

(2) $$b=\sqrt{m+n}+a$$

where *m* is the number of neurons in the input layer, *n* is the number of neurons in the output layer, and *a* is the integer between 1 and 10 [24].

#### 2.6.3. Optimal Wavelength Selection

The full spectra would suffer the risk of collinearity and redundant information, and the large amount of data of the full spectra would result in unstable, complex and poor performance models. Optimal wavelength selection would help reduce amount of data, computational task and redundant information, and build a simple and robust model.

The successive projections algorithm is a widely used variable method in spectral data analysis. SPA projects one variable on the other variables, and the variable with the largest projection is selected into the candidate subset of optimal variables. The multiple linear regression (MLR) method is used to select the final optimal variables from the candidate subset [25].

Variable importance in projection (VIP) is a variable selection method based on the PLS model. VIP score is an accumulation of importance of each variable to each component. The VIP score is defined as:

(3) vj=p∑a=1A[(qata'ta)(waj‖waj‖)2∑a=1A(qa2ta'ta)

where *a* =1, 2, …, A, *A* is the number of components (LVs), *w_aj_* is the loading weight for variable *j*, *q_a_* is the y-loading of the a th LV, *t_a_* is the scores of the ath LV. Variables with higher VIP score indicating greater importance could be selected as the optimal wavelengths. A threshold value is needed to select the optimal wavelength [26]. In this study, the threshold value was set as 1.21 [26].

Random frog coupled with PLS is a variable selection method which conducts random selection in an iterative manner. First, a subset of variables is initially selected, and for each iteration, the selected subset is updated. After N iterations, the selection probability of each variable is calculated. Then PLS models were built on the variables with the highest probability, and the variables with the lowest RMSECV of the corresponding PLS model were selected as the optimal variables [27].

### 2.7. Image Visualization of pH Distribution

Each pixel within the hyperspectral image contained a spectrum at the spectral range of the system. The chemical or physical features of each pixel could be predicted by the calibration models [17]. The general procedure of the image visualization was described as follows:

1. Extract the spectral data from the predefined ROI of the images segmented from the background.
2. Build a calibration model using the average spectra of the samples.
3. Select the optimal wavelengths and build the calibration model using optimal wavelengths. This procedure is optional.
4. Apply the calibration model on each pixel within the image to form a distribution map.

Image visualization by hyperspectral imaging provides the possibility to visual the chemical or physical features distribution within the sample and among the samples, which would be useful to monitor the biomass energy production process and production quality control.

### 2.8. Model Evaluation and Software

The models were evaluated by the correlation coefficient of calibration and prediction (*r_c_* and *r_p_*), and the root mean square error of calibration and prediction (RMSEC and RMSEP). The better calibration model should have high *r_c_* and *r_p_*, and low RMSEC and RMSEP. The hyperspectral image analysis was conducted on ENVI 4.6 (ITT, Visual Information Solutions, Boulder, CO, USA) and Matlab R2010b (The Math Works, Natick, MA, USA). The variable selection methods were conducted on Matlab R2010b. The BPNN models were built on Matlab R2010b. The PLS models were built on Unscrambler^®^ 10.1 (CAMO AS).

## 3. Results and Discussion

### 3.1. Spectral Features

**Figure 1:**
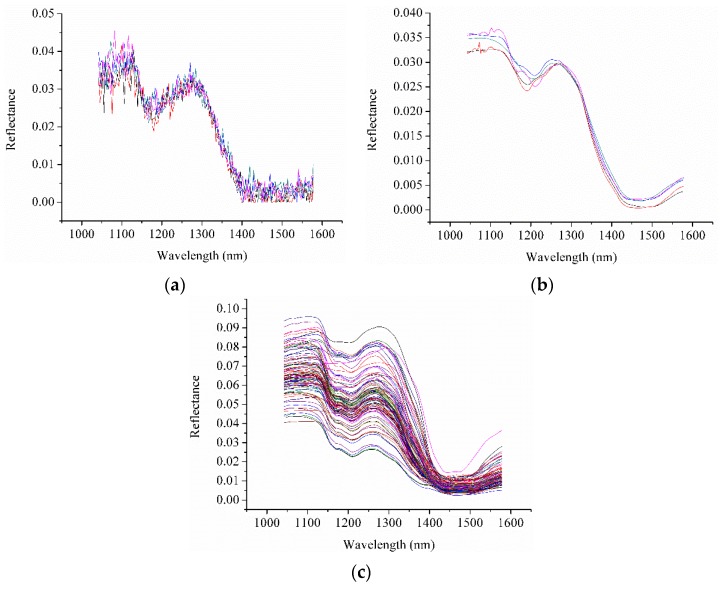
The unpreprocessed of spectra of (**a**) five randomly selected pixels; (**b**) the spectra of the five pixels preprocessed by WT; and (**c**) the average spectra of each sample.

Considering random noises caused by the imaging system, sample condition, and environmental factors, only the spectra in the 1042.16–1578.13 nm range were used. The spectra of five randomly selected pixels within a randomly selected ROI are shown in Figure 1a.

It was found that the unpreprocessed spectra were noisy. The blurs were obvious and randomly distributed. Only the general trend could be observed. The corresponding spectra preprocessed by WT with Daubechies5 (db5) and decomposition level of 5 (Figure 1b) were much smoother, with fewer or no blurs and maintaining the same spectral trend. The preprocessed spectra of pixels were similar to the average spectra of each sample (Figure 1c). The results indicated that WT method could be used to efficiently reduce the noise of the pixel spectra.

### 3.2. Split of Sample Sets

The samples were randomly divided into a calibration set and a prediction set at the ratio of 2:1. The statistical descriptions of the calibration set and the prediction set is shown in Table 1. The range of pH values of the prediction set was covered in the calibration set.

### 3.3. Calibration Models on Full Spectra

The average spectrum of each sample was used to build the PLS models and BPNN models. The results are shown in Table 2. The optimal numbers of LVs of the PLS model and neurons in the hidden layer of the BPNN model were 10 and 14, respectively. The PLS model gave good performance, with *r_p_* of 0.880 and RMSEP of 0.0695. The nonlinear BPNN model with *r_p_* of 0.894 and RMSEP of 0.0684 performed a little better than the linear PLS model. The results indicated that hyperspectral imaging could be used to determine the pH values during the anaerobic digestion for methane production.

### 3.4. Optimal Wavelength Selection

SPA, RF and VIP were used to select the optimal wavelengths. To select optimal wavelengths by SPA, the number of optimal wavelengths was set as 5 to 30. Finally, eight optimal wavelengths were selected. To select optimal wavelengths by RF, the number of iterations was set as 10,000, and 15 optimal wavelengths were finally selected. The selection of optimal wavelengths by VIP was based on the corresponding PLS model. The PLS model on the full spectra showed good performance. Thus, VIP could be used in this study. The threshold value used in this study was 1.21, as suggested by [26]. In total, 20 optimal wavelengths were selected by VIP. The selected optimal wavelengths are shown in Table 3. The selected optimal wavelengths were different due to the different selection methods used. The general methods for selecting optimal wavelengths at present have more mathematical meaning than utility in revealing the internal features of the spectra, resulting in different wavelengths being selected by different methods.

### 3.5. Calibration Models on Optimal Wavelengths

The PLS models and the BPNN models were built using the optimal wavelengths selected by SPA, RF and VIP. The results are shown in Table 4. For PLS models, the results were all acceptable with *r_c_* and *r_p_* over 0.8. SPA-PLS model obtained the best results, with *r_p_* of 0.853 and RMSEP of 0.0697.

**Figure 2:**
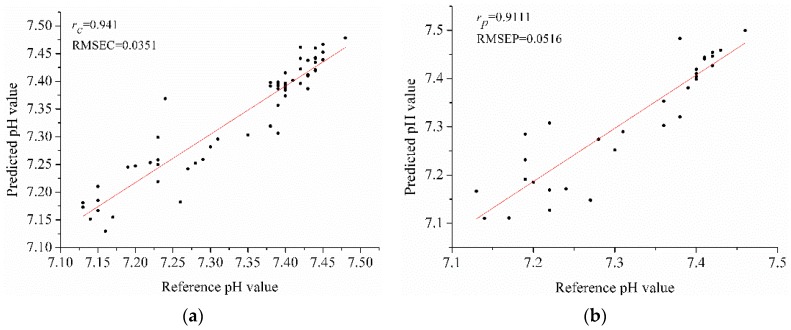
The results of SPA-BPNN model (**a**) calibration set; (**b**) prediction set.

For BPNN models, the results were all acceptable with *r_c_* and *r_p_* over 0.8. SPA-BPNN model performed best, with *r_p_* of 0.911 and RMSEP of 0.0516 (shown in Figure 2). The results indicated that the selected optimal wavelengths could be used to build PLS models and BPNN models for pH value determination during anaerobic digestion.

BPNN models performed slightly better than the corresponding PLS models. The reason might be that there was nonlinear information in the selected optimal wavelengths. SPA performed better than RF and VIP in PLS models and BPNN models.

Compared with the full spectra PLS model, PLS models using optimal wavelengths performed slightly worse. The number of wavelengths decreased by 95%, 90.625% and 87.5% in the SPA-PLS, RF-PLS and VIP-PLS models, respectively, which sped up the modeling procedure and simplified the models. The performance of *r_p_* were decreased at most 6.59% (VIP-PLS).

Compared with full spectra BPNN model, SPA-BPNN model performed slightly better and RF-BPNN and VIP-BPNN performed slightly worse. The results showed that variable selection methods were effective in pH determination by hyperspectral imaging and SPA was much more suitable in this study.

### 3.6. Image Visualization of pH Ddistribution

**Figure 3:**
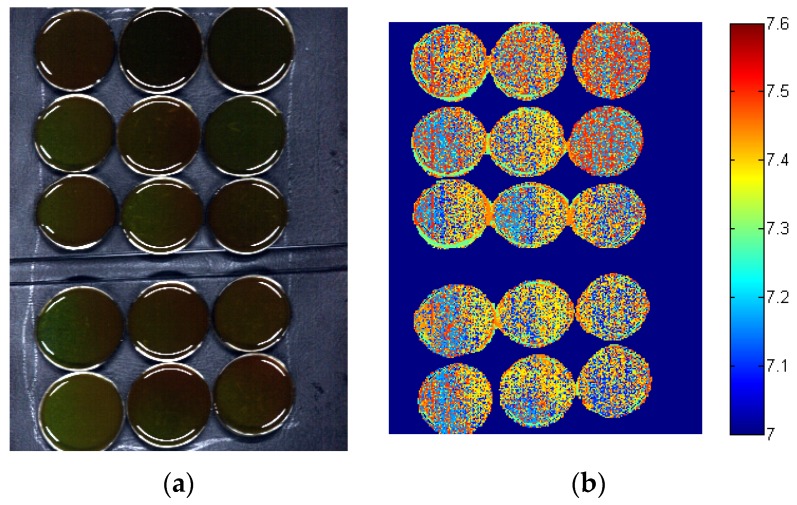
The pseudo color image of (**a**) a hyperspectral image and (**b**) the corresponding distribution map of pH obtained by SPA-BPNN.

As discussed above, BPNN models performed better than PLS models, and the SPA-BPNN model provided the best performance. The SPA-BPNN model was next applied to predict the pH value of each pixel and form the distribution map. The pseudo color image and the corresponding distribution maps were shown in Figure 3. It was found that most of the pixels were predicted in the range of the calibration set. The edge of the Petri dish had different spectral features from the samples, and it was observed that the prediction values of Petri dish edge were different from the prediction values of the sample regions.

The prediction values of some pixels were beyond the calibration set, the reason might be that the calibration set did not cover all the features of all pixels. It was quite difficult to satisfy the requirement of all features in the calibration set. An important issue to be emphasized was that it was quite difficult to test the accuracy of the prediction value of each pixel, because the reference pH value of the pixel was unknown and quite difficult to obtain. A robust and accurate calibration model covering the spectral features of all the pixels would help improve the accuracy of the prediction value. The general trend and the range of the prediction values were generally used to evaluate the predicted distribution map [19,28,29]. The results indicated the feasibility of using hyperspectral imaging to monitor reactions and parameters of every part within the anaerobic digestion liquids during dynamic process. A robust and accurate calibration model covering the widest sample range and more spectral features should be developed for more accurate prediction, which needed much more studies.

The hyperspectral imaging method showed great advantages over traditional pH meters and pH sensors, including fast, large area real time measurement, on-line monitoring, intelligent and automatic control. However, using hyperspectral imaging to monitor the pH value and the other parameters during the anaerobic digestion also has some drawbacks: (1) large primary input-cost. The hyperspectral imaging system is expensive, and developing a hyperspectral system required a great investment; (2) Model establishment and maintenance. The prediction model was very important in applying hyperspectral imaging. How to build robust and accurate model was an essential issue of concern. The model mantainance should also be studied to calibrate model parameters to meet the demands of different measurement situations and ensure the model robustness and accuracy. With the further development of science and technology, the price of a hyperspectral imaging system would go down, and the model establishment and maintenance would become easier. In all, hyperspectral imaing was a promising method for on-line monitoring of pH values during anaerobic digestion for biomass energy production.

## 4. Conclusions

The results showed that hyperspectral imaging combined with chemometric methods and variable selection methods could be used for the determination of pH values during anaerobic digestion for methane production. SPA-BPNN models presented the best performance. The SPA-BPNN models were used to obtain the distribution map. This study provides a new alternative to monitor the pH value during anaerobic digestion. Knowing the pH status of different locations of anaerobic digestion liquids would help to guide automatic control of pH for anaerobic digestion. Hyperspectral imaging could also be used to determine and visualize other parameters, providing guides for the automatic control for better methane production.

## Figures and Tables

**Table 1:** The statistic description of sample sets split.

Sample Set	Number	Range	Mean	STD ^a^
Calibration set	62	7.13–7.48	7.34	0.10
Prediction set	31	7.13–7.46	7.31	0.10

**^a^** STD = standard deviation.

**Table 2:** The results of PLS model and BPNN model on full spectra.

Models	LVs ^a^/Nodes	Calibration		Prediction	
		*r_c_*	RMSEC	*r_p_*	RMSEP
PLS	10/	0.904	0.0443	0.880	0.0695
BP	/14	0.910	0.0446	0.894	0.0684

**^a^** LVs were the number of latent variables in PLS model; nodes were the number of nodes in the hidden layer of BPNN mode.

**Table 3:** The optimal wavelengths selected by SPA, RF and VIP.

Methods	Number	Optimal Wavelengths (nm)
SPA	8	1210.29, 1395.67, 1129.54, 1287.75, 1058.95, 1574.74, 1520.64, 1372.05
RF	15	1378.8, 1274.27, 1183.36, 1237.22, 1240.59, 1270.91, 1301.23, 1375.42, 1277.64, 1129.54, 1176.63, 1109.36, 1159.8108, 1095.92, 1388.92
VIP	20	1042.16, 1045.52, 1156.4399, 1159.8108, 1163.9, 1166.54, 1203.55, 1206.92, 1210.29, 1213.65, 1217.02, 1355.1801, 1358.55, 1361.9301, 1365.3, 1395.67, 1399.04, 1402.42, 1405.79, 1409.17

**Table 4:** The results of PLS models and BPNN models on optimal wavelengths selected by SPA, RF and VIP.

Methods	PLS					BP				
	LVs	Calibration		Prediction		Nodes	Calibration		Prediction	
		*r_c_*	RMSEC	*r_p_*	RMSEP		*r_c_*	RMSEC	*r_p_*	RMSEP
SPA	7	0.891	0.0471	0.853	0.0697	6	0.941	0.0351	0.911	0.0516
RF	11	0.852	0.0545	0.829	0.0698	10	0.903	0.0463	0.877	0.0589
VIP	12	0.866	0.0519	0.822	0.0745	5	0.921	0.0417	0.820	0.0636
